# Editorial: Mucosal immunity after vaccination

**DOI:** 10.3389/fimmu.2025.1690399

**Published:** 2025-09-05

**Authors:** Mohd Arish, Mireia López-Siles, Farha Naz

**Affiliations:** ^1^ Beirne B. Carter Center for Immunology Research, University of Virginia, Charlottesville, VA, United States; ^2^ Microbiology of Intestinal Diseases, Biology Department, Universitat de Girona, Girona, Spain; ^3^ Department of Medicine, Division of Infectious Diseases and International Health, University of Virginia, Charlottesville, VA, United States

**Keywords:** mucosal immunity, vaccination, secretory IgA, mucosal vaccines, immune response, delivery systems, adjuvants, gut microbiome

The mucosal immune system is the largest immunological interface with the environment in our body. On the one hand, it contributes to the preservation of commensal microbiota by several mechanisms such production of secretory immunoglobulins, maintenance of the mucus layer, mucosal cell signaling -via production of cytokines, and chemokines-, or tolerance via regulatory T-cells, among others. On the other hand, the mucosal immune serves as both a barrier and an active immunological network that protects against pathogen invasion at entry sites. This Research Topic “*Mucosal Immunity after Vaccination*” in *Frontiers in Immunology* brings together six studies spanning human health, veterinary medicine, and experimental vaccine platforms. Collectively, it illustrates how diverse strategies from metabolic optimization to innovative delivery systems can converge on the goal of robust mucosal immunity after vaccination.

## Glycemic control and mucosal immune responses after vaccination in diabetes

Mojaddidi et al. review the effects of glycemic control on systemic and mucosal immunity in diabetic patients, demonstrating how chronic hyperglycemia compromises epithelial barrier integrity, reduces secretory IgA production, and impairs tissue-resident memory T cell activity. Their findings resonate with Yan et al., who highlight the importance of intact barrier function for effective mucosal responses in pigs, and Bhimani et al., who show that even with a novel delivery vehicle such as extracellular vesicles, host immune competence remains critical in mice. Similarly, in COVID-19, type 2 diabetes patients with poor glycemic control show weaker immune responses and more breakthrough infections after receiving vaccination with mRNA-BNT162b2. In contrast, those with HbA1c < 7% mount stronger neutralizing antibody and CD4^+^ T-cell/cytokine responses than poorly controlled patients (HbA1c ≥ 7%) (1).

## Vector-based mucosal vaccination in veterinary medicine

Yan et al. developed four recombinant swine acute diarrhea syndrome coronavirus (SADS-CoV) vectors expressing porcine epidemic diarrhea virus (PEDV) antigens fused with immune cell–targeting peptides. The constructs remained genetically stable for over 20 passages, and the recombinant viruses were capable of proliferating similarly to the wild-type virus in cell culture and maintained their structural characteristics. When administered to pregnant sows, it elicited strong mucosal and systemic responses that protected neonatal piglets from PEDV challenge. This maternal-neonatal protection model echoes other evidence that mucosal immunization in dams can transfer effective lactogenic immunity to offspring (2, 3). Importantly, the immune-cell targeting strategy parallels the dendritic cell-targeted minicell approach by Yang et al. using mice models, and demonstrates the broader principle that rational vector design, whether viral, bacterial, or vesicular, can significantly enhance antigen uptake and mucosal immunity.

## Extracellular vesicles as an oral vaccine platform

Nontyphoidal *Salmonella* (NTS) remains a leading cause of foodborne illness globally, responsible for significant morbidity and mortality, particularly in young children and immunocompromised individuals (4, 5). Despite the availability of licensed vaccines against *Salmonella typhi*, no approved vaccines exist for NTS, which is concerning given the pathogen’s increasing multidrug resistance (6). Innovative strategies are therefore urgently needed, and mucosal vaccination has gained attention because of its ability to induce both local and systemic immunity at the intestinal barrier; the primary site of pathogen invasion (7). Extracellular vesicle (EV) based vaccines have recently emerged as a promising approach, as EVs naturally package microbial antigens in immunogenic forms (8). Previous work demonstrated that intranasal delivery of EVs derived from *Salmonella*-infected macrophages conferred protection against lethal challenge in mice (9). The current findings by Bhimani et al. demonstrate that orally delivered small extracellular vesicles (sEVs) from *Salmonella*-infected macrophages protect mice from lethal *Salmonella typhimurium* challenge, reduce tissue bacterial burden, and stimulate antigen-specific IgG.

## Therapeutic mucosal vaccination against HSV-2

Quadiri et al. present compelling preclinical evidence using guinea pigs that adenovirus-based vaccines expressing human herpes simplex virus type 2 (HSV-2) ribonucleotide reductase 2 and glycoprotein D elicit robust tissue-resident CD4^+^ and CD8^+^ T cells in dorsal root ganglia and vaginal mucosa, leading to marked reductions in viral shedding and recurrent genital lesions. These findings are significant because recurrent genital herpes remains a major global health challenge with no licensed vaccine despite decades of effort (10). Previous vaccine strategies, including subunit glycoprotein D formulations, have failed to protect in clinical trials (11), highlighting the importance of local tissue-resident immunity. By leveraging adenovirus vectors to drive durable mucosal T-cell responses, this study offers a promising path toward therapeutic vaccines for genital herpes.

## Gut microbiota and vaccine efficacy against respiratory pathogens

In their timely review, Xue et al. synthesize growing evidence that the gut microbiota critically modulates vaccine responses to respiratory pathogens, including influenza and SARS-CoV-2. The gut-lung axis allows commensals to influence systemic immunity, shaping antibody titers, T-cell responses, and vaccine durability (12). Clinical studies have linked microbiota composition with differential antibody responses to influenza and COVID-19 vaccines (13, 14), while preclinical work shows that microbiota disruption impairs protective immunity (15). This perspective underscores the potential of microbiota-targeted interventions to enhance next-generation respiratory vaccines.

## *Salmonella* minicell-based vaccine against *Helicobacter pylori*

Yang et al. developed two innovative vaccine candidates based on engineered *Salmonella* minicells against *H. pylori*. These minicells consist of small non-dividing nanoparticles (100~500 nm) that arise from bacteria due to premature septation during cell division. As they can be engineered to package recombinant proteins or plasmid DNA, they are highly versatile for drug and protein delivery. Specifically, a multi-epitope vaccine was developed, including both B- and T-cell epitopes that elicited strong systemic and mucosal immunity against *H. pylori*, reducing bacterial colonization and gastric pathology *in vivo* when tested in mice. Specifically, the candidate coated with dendritic cell-targeting RNA aptamer to enhance antigen delivery and immune activation demonstrated superior immunogenicity, characterized by greater reductions in gastric bacterial loads, enhanced T-cell cytokine production, and increased mucosal IgA levels. This is important because *H. pylori* remains a major risk factor for gastric cancer, yet current antibiotic therapies are undermined by rising resistance (16). Multi-epitope vaccine strategies have shown promise in eliciting protective Th1/Th17 responses against *H. pylori* (17), while bacterial minicells represent a versatile platform for safe antigen delivery (18). By combining targeted delivery with epitope-based design, this study advances the development of next-generation vaccines for gastric pathogens.

Together, these studies underscore how innovative approaches across human, animal, and microbial systems are redefining the frontiers of mucosal vaccinology, paving the way for more effective and durable protection against diverse pathogens. However, differences regarding mucosal immunity exist among species and should be considered prior to translate these findings into clinical practice.
